# Cognitive-Behavioral Therapy Versus Transcranial Direct Current Stimulation for Augmenting Selective Serotonin Reuptake Inhibitors in Obsessive-Compulsive Disorder Patients

**DOI:** 10.32598/bcn.11.1.13333.2

**Published:** 2020-01-01

**Authors:** Mohsen Dadashi, Vida Yousefi Asl, Yousef Morsali

**Affiliations:** 1. Department of Clinical Psychology, Social Determinants of Health Research Center, University of Medical Sciences, Zanjan, Iran.; 2. Department of Psychology, Zanjan University of Medical Sciences, Zanjan, Iran.

**Keywords:** Exposure and Response Prevention (ERP), Transcranial Direct Current Stimulation (tDCS), Obsessive Compulsive Disorder (OCD)

## Abstract

**Introduction:**

Obsessive-Compulsive Disorder (OCD) belongs to the categories of psychiatric disorders with the potential to turn into a chronic condition without receiving the necessary treatments. The main feature of OCD is the frequent or intense obsession and compulsion that might induce great pain and suffering in patients. Moreover, as one of the most prevalent abnormalities, depression usually follows OCD. The present study aimed to compare the effects of Exposure and Response Prevention (ERP) and Transcranial Direct Current Stimulation (tDCS) treatments adjunct to pharmacotherapy on decreasing the severity of obsession-depression symptoms and improving the quality of life in OCD patients.

**Methods:**

This was a quasi-experimental study with a pre-test, post-test design and a follow-up stage. The statistical population comprised all the patients diagnosed with OCD in Zanjan Province, Iran. Besides, 26 OCD patients referring to Shahid Beheshti Medical Center in Zanjan were selected using a purposive sampling method. Then, they were randomly assigned to two treatment groups. The study subjects completed the Yale Brown Obsessive-Compulsive Scale (Y-BOCS), Beck Depression Inventory-II (BDI 2), and the Quality of Life Questionnaire at the pre-treatment, post-treatment, and follow-up stages (1 month and 2 months after the treatment). Analysis of Covariance (ANCOVA) and Reliable Change Index (RCI) methods were used to measure statistical and clinical significances, respectively. The collected data were analyzed using SPSS.

**Results:**

The obtained data suggested no significant difference between the ERP and tDCS groups concerning the symptoms of OCD and depression at the post-test stage (P>0.05). Conversely, in terms of life quality, there was a significant difference between the ERP and tDCS groups at the post-test phase (P<0.05).

**Conclusion:**

Although the present findings revealed no statistically significant difference between the ERP and tDCS groups (except for the quality of life variable), the pharmacotherapy-ERP combination proved to be more effective than pharmaco therapy-tDCS in treating OCD patients.

## Highlights

- OCD is apt to be categorized as psychiatric disorders with the potential to turn into a chronic condition.
- There was a significant difference between the ERP and tDCS groups at the post-test stage.
- The pharmacotherapy-ERP combination proved to be more effective than pharmaco therapy-tDCS in treating OCD patients.

## Plain Language Summary

OCD is known as a mental disorder distinguished by the frequent or intense obsession and compulsion that cause great pain, which not only disturb normal life, work performance, routine social activities, and interpersonal relationships, but also waste OCD patient’s valuable time. Diagnosing the mental health disorders was conducted based on the SCID, and DSM-IV. Several treatments are suggested for OCD, including psychodynamics, behavioral therapy, cognitive therapy, CBT, pharmacotherapy, supportive psychotherapy, group therapy, family therapy, electroconvulsive therapy, deep brain stimulation, and tDCS. By controlling the pre-test effect, the ANCOVA results indicated no significant difference between the ERP and tDCS groups concerning the symptoms of OCD and depression at the post-test and both follow-ups. The evaluation of the study subjects’ scores in ERP and TDCS groups revealed that both methods have been effective in treating OCD when combined with pharmacotherapy. The clinical effectiveness of the ERP method was more significant.

## 1. Introduction

Obsessive Compulsive Disorder (OCD) is generally defined as a mental disorder distinguished by the presence of frequent or intense obsession and compulsion that cause great pain and suffering for the patients. Furthermore, obsession and compulsion not only disturb normal life, work performance, routine social activities, and interpersonal relationships, but also waste OCD patient’s valuable time. An OCD patient may suffer from obsession, compulsion, or both. OCD ranks the fourth among the most prevalent psychiatric disorders following panic disorder, substance use disorder, and major depressive disorder. This mental disorder disrupts different life dimensions and is accompanied by the exacerbation of symptoms (Sadock, 2014).

Several treatments are suggested for OCD, including psychodynamics, behavioral therapy, cognitive therapy, Cognitive-Behavioral Therapy (CBT), pharmacotherapy, supportive psychotherapy, group therapy, family therapy, electroconvulsive therapy, deep brain stimulation, and Transcranial Direct Current Stimulation (tDCS). The odds of OCD improvement without applying any treatment has proved to be low (20% in a reassessment after 40 years) (Association, 2013). Pharmacotherapy and Exposure and response Prevention (ERP) are effective in treating OCD (Steketee & Schemmel, 2006); however, complete recovery has not been achieved in some patients for whom antipsychotics or CBT, including ERP were recommended as alternative treatments (Abramowitz, Foa, & Franklin, 2003; Simpson, Liebowitz, & Huppert, 2013). Previous evidence indicated that OCD is largely influenced by biologic factors; some malfunctions were observed in some brain parts (Sadock, 2014). OCD is a prevalent disease and, in some cases, desirable progresses have been achieved in treating patients. However, 30%–60% of the patients fail to tolerate the adverse effects of medications or at best present a relative response to the treatments combined with CBT. There is mounting evidence supporting that the following neurobiological components were involved in the OCD outbreak: abnormal activity and communication in orbitofrontal-striato-pallido-thalamic networks, increased activity in orbitofrontal, Supplementary Motor Area (SMA), cingulate gyrus, caudate nucleus, and reduced activity in the right and left cerebellum as well as parietal lobe. The tDCS is a non-invasive method that can be used to improve brain imbalance and brain-circuit communication. This method has not been sufficiently studied in spite of its advantages in empowering the hospital staff as well as the therapists working in this field. Therefore, it is necessary to conduct research projects to evaluate the effectiveness of this treatment; accordingly, we could help the patients who have not positively responded to the current treatments (Bation, Heasebeart, Saoud, & Brunelin, 2016).

## 2. Methods

This study employed a clinical trial that was approved by the Ethics Committee of Zanjan University of Medical Sciences on December 15, 2016 involving the following Codes: ZUMS.REC.1394.336 and IRCT and IRCT2016061728504N1.

The study sample consisted of all patients diagnosed with OCD in Zanjan Province, Iran. Moreover, 26 OCD patients referring to Shahid Beheshti Medical Center in Zanjan were selected using Diagnostic and Statistical Manual of Mental Disorders, 5^th^ Edition (DSM-5) criteria and purposive sampling technique. The study participants were randomly assigned into two treatment groups.

To meet the inclusion criteria, the study subjects must have demonstrated the OCD symptoms confirmed by a psychiatrist based on a diagnostic interview and a clinical psychologist based on the Structured Clinical Interview for DSM-IV Axis I Disorders (SCID-I). They should have had ≥3rd grade middle school education with an age range of 18–50 years. Finally, the study participants needed to complete the written consent form and not to be exposed to any psychological and complementary therapies for ≥1 month before entering the research.

The exclusion criteria consisted of having suicidal or homicidal thoughts, complete symptoms of personality disorder, and psychotic disorders. Additionally, those who missed >2 sessions of the treatment meetings and failed to do any homework were excluded from the study. In this research, the following questionnaires were used for data collection.

### 2.1. Structured Clinical Interview for DSM Disorders (SCID)

Diagnosing the mental health disorders was conducted based on the SCID, and the Diagnostic and Statistical Manual of Mental Disorders, Fourth Edition (DSM-IV). The follow-up interview included a flexible, comprehensive, and standard inventory developed. Tran and Smith reported a 60% Cohen’s kappa coefficient (K) as the validity coefficient among the raters of SCID. After translating the interview into Persian, Sharifi et al. (2008) conducted this interview on a sample of 299 individuals. The diagnostic agreement was analyzed to range from medium to good (K>60%) for which the overall agreement was satisfactory (total Kappa diagnosis= 52% and the total lifetime diagnosis=55%) (Group, 1998).

### 2.2. Yale-Brown Obsessive-Compulsive Scale (YBOCS)

The 10-item Y-BOCS scale was employed to measure the severity of OCD symptoms without being biased towards the content of obsessions or compulsions. In the Y-BOCS scale, 5 out of 10 items focusing on obsessive thoughts and the rest on compulsive behaviors. The inter-rater reliability of this scale was evaluated to be 0.98 based on its administration among 40 patients. The internal consistency coefficient was found to be 0.9, and its 2-week interval test-retest reliability was reported to be 0.84. The preliminary examination phase, which was performed on 26 patients to examine the convergent validity; accordingly, the correlation between the scores of this scale and the Maudsley Obsessional-Compulsive Inventory (MOCI) was found to be 0.72.

### 2.3. Beck Depression Inventory (BDI-2)

This tool is a revised version of the Beck Depression Inventory, which aims to measure depression severity. The present version is more consistent with DSM-IV, compared to its first edition and covers all depression elements which are rooted in cognitive theory. Beck, Steer, and Brown’s findings have supported the high internal consistency of this questionnaire. The internal consistency, as well as the test-retest reliability of it, were found to be 0.87 and 0.73, respectively. In a sample of 94 people in Iran, Fata, Birashk, Atef-vahid, & Dobson (2011) reported the Cronbach’s alpha coefficient of 0.92 and a one-week test-retest reliability of 0.9 for this tool. Respondents are required to have the reading ability of a 5^th^ or 6^th^ grader for understanding the questionnaire’s items. They must respond to each item based on a 4-point Likert-type scale ranging from 0 to 3 in which the minimum and maximum scores are considered to be zero and 3, respectively.

### 2.4. Personal Wellbeing Index-Adult (PWI-A)

The PWI-A was developed by Cummins and La in 2006 as per the International Welfare Group’s request. The core set of items forming PWI-A comprises 7 questions of satisfaction assay, including specific life domains as follows: living standards, personal health, life accomplishments, personal relationships, personal safety, community connectedness, and future security. These items are integrated into the general question of “how satisfied are you with your life as a whole?” To answer the questions, the person specifies the satisfaction level of each item on a scale varying from zero to 10. On this scale, zero indicates no satisfaction at all, and 10 demonstrates complete satisfaction. In the case of Australian contexts, Cronbach’s alpha coefficient has been reported to be ranging from 0.70 to 0.85 (Group, 2006).

The present study used a quasi-experimental design involving pre-test, post-test and follow-up stages. The study sample consisted of 26 OCD patients referring to the psychiatric clinic of Shahid Beheshti Medical Center in Zanjan City, Iran. These patients were diagnosed with OCD by a psychiatrist and entered the study after the initial evaluation. Then, the study subjects were randomly assigned to two treatment groups of ERP (n=13) and tDCS (n=13). Each group was evaluated 4 times as follows: before the intervention (pre-test), after the intervention (post-test), one-month follow-up, and two-month follow-up. These evaluations were conducted by a person other than the researchers (who had an MA. in clinical psychology) working at the educational-health center. In each of the ERP and tDCS groups, three patients were excluded from the study during the treatment. In the one- and two-month follow-ups, one patient from the ERP and three from the tDCS groups, were excluded from the study.

Additionally, the treatment was performed individually, and in the case of the pharmacotherapy, the patients were treated under the supervision of a psychiatrist. Furthermore, the type and drug dosage were controlled by a psychiatrist to minimize the differences between the two groups. The treatment process was as follows. The first session in both treatment groups were dedicated to the OCD diagnostic interview, as well as the establishment of a proper treatment relationship and collection of information about the patient’s OCD. The BDI, Y-BOCS, and PWI-A questionnaires were administered at the pre-test. Next, an informed consent form, including treatment-related explanations, was provided to the patients. At the end of the treatment sessions, the questionnaires were completed as post-tests, then used in the follow-ups. In the treatment sessions, the study patients were provided with some information regarding the onset of OCD, the process of illness, treatments, and treatment logic. In the next section, we discussed the treatments implemented in each group.

In the SSRIs treatment (fluoxetine and fluvoxamine), the psychiatrist prescribed a standard dose of the medication for daily intake (between 40 and 60 mg for fluoxetine and between 100 and 200 mg for fluvoxamine). The treatment process was conducted in 10 sessions during which the patient was presented with some information on the logic of treatment, the effects of drugs, and instructions on how to use the drug. Eventually, the appropriate dose was prescribed for the patient. At subsequent sessions, the patient was examined to determine the effects and adverse effects of the treatment. Then, the patient was encouraged to use the medication regularly.

Patients received tDCS treatment for 10 days as follows. Each day, for 20 minutes, the study patients were treated using a flow of 2 mA, cathode electrodes in the orbitofrontal cortex (FP1), and anode on the right side of the cerebellum (Bation, Heasebeart, Saoud, & Brunelin, 2016). The direct flow was transmitted through the electrodes covered with a saline-soaked sponge. The size of the electrodes in this study was 4.5×4.4 cm.

In the ERP group, cognitive techniques were conducted from the third session. This method’s rationale was to acquaint the patient with cognitive distortions, debilitate (and not change) the obsessive beliefs, and prepare them for the ERP stage. Cognitive techniques rectify the misunderstandings about OCD and help the person to overcome the feelings of shame and anxiety caused by OCD symptoms. Cognitive strategies often target the distress caused by obsessive-compulsive thoughts and aim to undermine them. In general, reducing risk assessment, individual responsibility, and similar factors increased the patient’s participation in the ERP (Leahy & McGinn, 2012).

The EPR method was conducted at the beginning of the process. This method’s purpose was to debilitate the association between obsessive thoughts and negative emotions. In the present technique, the patients were instructed to understand that their anxiety and obsessive compulsions were reduced by being exposed to a regular confrontation with obsessive thoughts as well as the situations which provoke those thoughts without avoiding or undermining them.

At the first stage, the study patients were exposed to the situations that were less stressful for them under the supervision of the therapist. During exposure, the individual’s anxiety levels were continuously measured on the scale of zero to 10. Besides, their physical symptoms, such as heartbeat, body temperature, shortness of breath, dizziness, headache, and other anxiety symptoms, were examined. The exposure continued until the anxiety of the patient was significantly reduced where it reached its base level; before the onset of exposure or half of it. The first session of exposure to anxiety lasted about 90 minutes. Although getting accustomed to the treatment sessions was not considered as a crucial indicator of the treatment success, it was recommended to reduce the anxiety to its minimum level.

Then, the patient was prescribed to perform the exposure daily as homework until the same position triggered the least anxiety. When the patient successfully dominated the first stage of the hierarchy, the confrontation with the next anxiety level initiated. In other words, when the anxiety decreased within at least two consecutive sessions, the study participant was able to move to the next stage of the anxiety hierarchy. Response prevention directly targeted the obsessive-compulsive and avoidance behaviors of the patients and was applied in conjunction with exposure. In this technique, the patients were trained to reduce their avoidance behaviors through exposure to obsessive thoughts and finally eliminate them.

Consequently, they could withstand the anxiety caused by obsessive thoughts and natural stimulators. With the onset of exposure, the rules were set aside, and the patients were encouraged to internalize two lessons; the rules do not prevent the risk, and the obsessions are not dangerous. Thus, the patients needed to stop their rule governing behavior during the exposure and the day. Otherwise, they may have decided to use the rules after the exposure and consequently reduce their anxiety.

When all anxiety exposure and response prevention stages were practiced, the treatment sessions ended. Eventually, the patient was not anxious in anxiety-provoking situations and did not have any obsessive behavior. Treatment practices were implemented for preventing the recurrence of OCD and helping the patients to identify the potential sources of their anxiety. In the final session, BDI, Y-BOCS, and PWI-A questionnaires were completed by the study subjects as the post-tests. One- and two-month follow-ups were also conducted to evaluate the long-term efficacy of the treatment.

Analysis of Covariance (ANCOVA) and repeated-measures Analysis of Variance (ANOVA) were used to examine the effectiveness of the treatments in SPSS. The existence of significant statistical differences between the pre-test, post-test scores did not necessarily indicate an unsuccessful life of the patients. Therefore, it is necessary to use methods that complement the statistical tests and provide more precise results regarding the effectiveness of psychological therapies. In this respect, the contribution of reliable methods determining clinically significant changes would be of great help. In this study, the Reliable Change Index (RCI) (Formula 1) was applied to determine whether the resulting changes were reliable or merely occurred due to a measurement error:

1. $$\begin{array}{l} \text{RCI}=\left(\text{pre-test},\;\text{post-test}\right)/\surd {\left(2\;\text{SE}\right)}^{2}\\ \text{SE}=\text{SD}\surd \left(1-\text{r}\right) \end{array}$$

If the absolute value of the RCI index was >1.96, it could be concluded with 95% certainty that the observed pre-test, post-test score difference has not occurred incidentally; it rather indicates a stable and real change (Asghari Moghaddam, & Shairi, 2014).

## 3. Results

The demographic characteristics of the studied subjects are presented in Table 1. The Chi-squared test was used to compare the two groups of ERP and tDCS in terms of gender, marital status, education, and occupation. Considering the normal distribution of age based on the Kolmogorov-Smirnov test as well as the similarity of variances in the two groups based on the Levene’s test, an Independent Samples t-test was run. The Chi-squared test results revealed no significant difference between the two groups of ERP and tDCS in terms of gender, marital status, education, and occupation (Table 1).

**Table 1.** Demographic characteristics of subjects

**Variables**		**No. (%)**		**Chi Square Test Results**	

		**Groups**			

		**ERP**	**tDCS**	**X^2^**	**P**
sample		10 (100)	10 (100)		
Gender	Female	8 (80)	7 (70)	0.286	0.867
	Male	2 (20)	3 (30)		
Marital status	Single	4 (40)	5 (50)	0.202	0.653
	Married	6 (60)	5 (50)		
Education	intermediate	3 (30)	4 (40)	0.286	0.867
	High school	4 (40)	3 (30)		
	University	3 (30)	3 (30)		
Job	Housewife	6 (60)	5 (50)	0.291	0.865
	Employed	2 (20)	2 (20)		
	University Student	2 (20)	3 (30)		

**Variable**	**Mean±SD**		**Independent t-test**	

			**T**	**P**
Age	305±7.5	28.3±7.1	0.673	0.509

* P<0.01;

** P<0.05

Similarly, the Independent Samples t-test results suggested no significant difference between the two groups (Table 1). Therefore, the ERP and tDCS groups were homogeneous in terms of the demographic variables. Independent Samples t-test results in terms of the pre-test on dependent variables revealed no significant difference (P=0.5). In other words, at the pre-test stage, there was no significant difference between the ERP and tDCS groups in terms of obsessive-compulsive symptoms, depression, and life quality. The intergroup univariate ANCOVA was used to compare ERP and tDCS approaches by controlling the initial between-group differences at the pre-test stage, which was considered as the covariate.

By controlling the pre-test effect, the ANCOVA results indicated no significant difference between the ERP and tDCS groups concerning the symptoms of OCD and depression at the post-test and both follow-ups. Although the mean score for the ERP group at the post-test and follow-ups was more than that of tDCS; however, there was no statistically significant difference between the two groups. The effectiveness of ERP treatment in reducing OCD and depression was greater than that of tDCS (Table 2).

**Table 2.** Average, standard deviation and Covariance analysis

**Variable**	**Phase**	**Mean±SD**		**Source**	**df**	**Mean Square**	**F**	**P**	**Eta**

		**Groups**							

		**ERP**	**tDCS**						
OCD	Post-test	14.4±11.9	22.2±6.9	Group	1	196.5	3.04	0.099	0.152
				Error	17	56.6			
	Follow-up (1)	12.9±13.9	21.7± 11.5	Group	1	131.3	1.58	0.230	0.109
				Error	13	82.9			
	Follow-up (2)	13.5±11.4	20.7±9.7	Group	1	157.6	2.4	0.144	0.157
				Error	13	65.23			
Depression	Post-test	13.5±12.7	17.9±9.1	Group	1	85.16	1.08	0.314	0.060
				Error	17	79			
	Follow-up (1)	12.6±13.6	16.6±9.1	Group	1	0.221	0.003	0.958	0.000
				Error	13	76.49			
	Follow-up (2)	12.9±12.7	16.1±8.4	Group	1	1.12	0.016	0.903	0.001
				Error	13	71.8			
Quality of Life	Post-test	55.5±21.4	43.4±16	Group	1	995.2	5.99^*^	0.026	0.261
				Error	17	166.1			
	Follow-up (1)	56.6±26.1	43.6±19.9	Group	1	844.5	4	0.067	0.236
				Error	13	210.75			
	Follow-up (2)	56.5±26.2	45.1±16.7	Group	1	643.3	2.6	0.130	0.168
				Error	13	245.7			

* P<0.05

Average, standard deviation and Covariance analysis statistics for comparison of ERP group with tDCS in dependent variable: Obsessive-compulsive, depressive and quality of life symptoms

In the ERP and TDCS groups, the results of RCI effects on OCD presented significant clinical changes at pre-test, post-test, and follow-ups (Tables 3 & 4). The results of RCI’s impacts on depressive symptoms suggested that the ERP group did not illustrate any significant change from the pre-test to post-test; however, it significantly changed at the one- and two-month follow-ups (Table 3). In other words, the changes did not occur randomly and remained stable.

**Table 3.** Estimation of RCI (ERP group)

**Variable**	**Mean±SD**				**RCI**		

	**Pre-test**	**Post-test**	**Follow-up (1)**	**Follow-up (2)**	**Pre-Post**	**Pre-Follow-up (1)**	**Pre-Follow-up (2)**
OCD	27.1±4.8	14.4±11.9	13.9±12.9	13.5±11.4	4.67	5.33	5
Depression	27.6±10.3	13.5±12.7	13.6±12.6	12.9±12.7	1.86	1.98	1.94
Quality of Life	38.2±17.9	55.5±21.4	56.6±26.1	56.5±26.2	−1.37	−1.45	−1.45

Descriptive statistics and estimation of RCI in four time periods and their comparison in dependent variables (ERP group)

**Table 4.** Estimation of RCI (tDCS group)

**Variable**	**Mean±SD**				**RCI**		

	**Pre-test**	**Post-test**	**Follow-up (1)**	**Follow-up (2)**	**Pre-Post**	**Pre-Follow-up (1)**	**Pre-Follow-up (2)**
OCD	28.5±4.7	22.2±6.9	21.7±11.5	20.7±9.7	2.37	2.6	2.93
Depression	28±9.8	17.9±9.1	16.6±9.1	16.1±8.4	1.4	1.58	1.65
Quality of Life	40.5±13.4	43.4±16	43.6±19.9	45.1±16.7	−0.31	0.33	−0.46

Descriptive statistics and estimation of RCI in four time periods and their comparison in dependent variables (tDCS group)

In the tDCS group, concerning the depression variable, the RCI results were not clinically significant; the observed changes were not due to stable changes (Table 4). According to Table 2, by controlling the pre-test effects, the ANCOVA results indicated a significant difference between post-test scores of ERP and tDCS groups in terms of the quality of life. The ERP treatment was more effective in improving the patients’ quality of life at the post-test phase. Besides, there was no significant difference between the ERP and tDCS groups at one- and two-month follow-ups. Although the mean value for the ERP group at one- and two-month follow-ups was higher than that of the tDCS group, no statistically significant difference was found between the two groups.

Furthermore, there was no significant difference between the ERP and tDCS groups at one- and two-month follow-ups. Although the mean scores of the ERP group at one- and two-month follow-ups were more than that of the tDCS group, there was no statistically significant difference between the two groups. Based on the obtained data, the effectiveness of ERP treatment in improving the quality of life was more than that of the tDCS method.

## 4. Discussion

The evaluation of the study subjects’ scores in ERP and TDCS groups revealed that both methods have been effective in treating OCD when combined with pharmacotherapy. The clinical effectiveness of the ERP method was more significant.

Regarding the obsessive-compulsive symptoms, Alilu, Bakhshi Pour, and Farnam (2008), indicated a significant difference between the control group and CBT and “exposure and response prevention” groups in the reduction of OCD symptoms; this finding is consistent with that of the present study. The meta-analysis study by Asadi, Shiralipour, Shakuri, & Mohammadkhani (2012). revealed that CBT was effective in treating OCD (Asadi, Shiralipour, Shakuri, & Mohammadkhani, 2012). Abramowitz also argued that ERP treatment is associated with significant changes in reducing OCD symptoms (Abramowitz, Foa, & Franklin, 2003).

In line with the present study findings, in a study by Bation, Heasebeart, Saoud, & Brunelin (2016) which used tDCS treatment as a cathode on the left OFC and anode on the right side of the cerebellum, OCD symptoms were reduced. Furthermore, patients who were not involved in any pharmacotherapy did not respond to tDCS treatment. Therefore, the combination of SSRIs and tDCS could be more successful in treating purposes than any other method (Bation, Heasebeart, Saoud, & Brunelin, 2016). The significant effects of tDCS treatment on reducing the OCD symptoms were primarily due to its combination with pharmacotherapy. Kuo and Nietsche (2014) used tDCS as a cathode in the F3 region and anode in the shoulder; there was no reduction in OCD symptoms. The greater sample size in the present study can be the reason for this inconsistency. Additionally, the co-administration of tDCS and pharmacotherapy may have contributed to its effectiveness (Kuo, & Nitsche, 2014).

Abramowits indicates that ERP treatment is associated with significant changes in the reduction of depressive symptoms (Abramowitz, Foa, & Franklin, 2003). Zandberg, McLean, Yeh, Simpson, & Foa (2015) have argued that changes in OCD and depressive symptoms affected each other, and OCD improvement has a greater impact on depression than the other way around. Changes in OCD symptoms had an overall effect on treating depressive symptoms. The reduction of depressive symptoms, which has occurred in response to ERP treatment, may have reduced the OCD symptoms. This decrease appears to be due to the reduced symptoms of the main illness (Zandberg, et al. 2015). The results of a case study by Alizadeh Goradel, Pouresmali, Mowlaie, & Sadeghi Movahed (2016) demonstrated that tDCS significantly affected depressive and obsessive-compulsive symptoms (Alizadeh Goradel, et al. 2016). Aronson, Katnani, and Eskandar (2014) documented that the Dorsolateral Pre-frontal Cortex (DLPFC), Orbitofrontal Cortex (OFC), Anterior Cingulate Cortex (ACC), and corpus striatum acted as the most important brain structures related to depression, anxiety, and OCD; their cathodic stimulation leads to a decrease in the symptoms of depression, anxiety, and OCD (Aronson, Katnani, & Eskandar 2014).

Abramowitz, Foa, & Franklin (2003) investigated the effectiveness of ERP on depression symptoms in OCD patients who presented severe depression comorbidity. Their results were inconsistent with those of the present study. In patients who suffer from a co-morbid depressive disorder, decreased anxiety levels cannot be achieved due to intense and high emotional response. Furthermore, the treatment outcome (feeling comfortable in the next exposure to anxiety stimuli), as well as the understanding of the unrealistic nature of the patient’s obsessive-compulsive discomfort, may not be achieved (Abramowitz, 2006).

Nowadays, the quality of life is regarded as an essential health aspect which has been measured in various studies. To the researchers’ best knowledge, there is no separate and independent study regarding the effect of tDCS treatment on the quality of life. However, in line with the findings of the present study, the results Sirvastava study suggested a significant improvement in the quality of life before and after conducting the CBT (especially ERP); there was also a changed effect size from pre-test to post-test concerning life quality. Furthermore, there was a significant relationship between Y-BOCS and the quality of life scores at post-treatment and follow-up stages (Srivastava, 2008). Other studies have also indicated that improved quality of life in clients who have responded to treatment was higher than that of those who did not. In other words, as a result of the recovery from symptoms, the burden of the disease will decrease in the individuals’ daily lives (Moritz, Fricke, Karow, Morfeld, Jelinek, & Jacobsen, 2005).

Contrary to the results mentioned above, there was no significant relationship between the severity of OCD symptoms and quality of life in the study of Speisman (2012). Vasudev and Saya (2015) investigated OCD and found that the quality of life only improved in the psychosocial dimension. Findings that indicated improvement in the quality of life were considered as the sub-categories of OCD treatment, such as pharmacotherapy, CBT, tDCS, and Deep Brain Stimulation (DBS) (Soh, Vaingankar, Picco, & Chong, 2013).

The following factors are among the limitations of this project that should be taken into consideration when interpreting the results: first, the sample was selected from a single institution, namely Shahid Beheshti Medical Center of Zanjan. Second, the size of the sample, as well as the study participants’ differences in number of the years of disorder development, were the other limitations of this study. The implementation of similar studies with a larger number of participants can increase the generalizability of these findings. It is also suggested to base tDCS treatment protocol on every individual’s specific brain map and Quantitative Electroencephalography (QEEG) to identify and treat the involved brain regions.

The obtained results should be interpreted with great caution. Although the researchers attempted to control the situation at most possible, it is difficult to control all the conditions regarding psychological treatment in the case of all human subjects. Based on the study findings, ERP and tDCS treatments, along with pharmacotherapy, could significantly reduce the symptoms of OCD and depression among patients from pre-test to post-test and follow-ups. Moreover, they may help improve the patients’ quality of life. In this study, the mean scores for this change in the ERP group were higher and found to be more effective in this regard. Therefore, it is advised to use this treatment along with medications in treating OCD patients. Further studies are requested to address the suggestions and limitations of this study.
